# Trehalose improves cold tolerance of *Pediococcus pentosaceus* OL77 and enhances low-temperature oat silage fermentation

**DOI:** 10.1128/msphere.00169-26

**Published:** 2026-04-30

**Authors:** Jikuan Chai, Chaosheng Liao, Zeliang Ju, Xin Liu, Xinyi Qu, Jie Bai, Guiqin Zhao

**Affiliations:** 1 Pratacultural College, Gansu Agricultural University 74661, Lanzhou, China; 2 Qinghai Academy of Animal Husbandry and Veterinary Sciences, Qinghai University 207475, Xining, China; Utah State University, Logan, Utah, USA

**Keywords:** trehalose, *Pediococcus pentosaceus*, cold adaptation, silage fermentation, compatible solutes

## Abstract

Low temperature remains a major bottleneck in silage fermentation, especially in cold regions and high-altitude areas. This study investigated the regulatory role of compatible solutes in enhancing the cold adaptation of *Pediococcus pentosaceus* OL77 and their synergistic application in oat silage under suboptimal temperatures (5°C–15°C). RT-qPCR analysis showed that expression of the cold shock protein gene *CspP* was sharply induced at 5°C, indicating its central role in cold-stress response. Exogenous trehalose and betaine significantly downregulated *CspP* expression, promoted bacterial growth, accelerated acid production, and enhanced metabolic stability. In silage trials, the OL77 + trehalose treatment resulted in improved lactic acid production, reduced ammonia nitrogen accumulation, and suppression of yeasts and molds across all tested temperatures. A random forest model identified 11 core variables critical for silage quality, highlighting the combined treatment as the most effective across all temperature conditions. These findings offer a robust theoretical and practical framework for enhancing low-temperature silage through synergistic application of compatible solutes and psychrophilic lactic acid bacteria and demonstrate the potential of machine learning in fermentation process optimization.

**IMPORTANCE:**

Low temperature is a major constraint on silage fermentation in cold and high-altitude regions. This study shows that trehalose improves the cold adaptation and fermentation performance of *Pediococcus pentosaceus* OL77, highlighting a practical strategy for improving oat silage quality under suboptimal temperatures.

## INTRODUCTION

During silage fermentation, low temperature is a frequent environmental constraint, especially in cold seasons and high-altitude regions. As the core functional microbial group, lactic acid bacteria (LAB) drive silage fermentation, and their activity directly determines preservation efficacy and nutritive value. However, lactic acid bacteria are highly sensitive to environmental fluctuations, and stress adaptation is largely governed by gene-expression regulation (1). Under low-temperature ensiling, lactic acid bacteria often face concurrent stresses from cold and progressive acidification, which together reshape cellular homeostasis and fermentation kinetics (2–6). A mechanistic understanding of how lactic acid bacteria respond to cold stress at the gene-expression level can therefore support the rational improvement of low-temperature inoculants (7, 8). Notably, recent silage-focused syntheses emphasize that inconsistent silage outcomes remain a persistent bottleneck and highlight modern biotechnology approaches, including transcriptomics, as essential for identifying and developing high-performance strains rather than relying on endpoint traits alone (9).

Under low-temperature conditions, bacteria typically show a canonical cold shock response in which global translation is repressed, while cold shock proteins are rapidly induced and function as RNA chaperones that sustain gene expression and protein synthesis at low temperature (10, 11). In *Escherichia coli*, the abundance of *CspA* is almost negligible at 37°C but increases dramatically following a shift to 10°C, and multiple homologs collectively form the *Csp* family (12, 13). Lactic acid bacteria harbor analogous *Csp* proteins. Importantly, in *Lactococcus lactis*, genetic and proteomic evidence shows that cold shock proteins contribute to cryoprotection and to the production of cold-induced proteins (14), supporting the use of *Csp* transcriptional dynamics as a mechanistic readout rather than a descriptive marker.

In parallel with cold shock regulation, bacteria must maintain membrane function and macromolecular stability to preserve solute exchange and osmotic balance during stress. This requirement is frequently met through accumulation of compatible solutes, including glycine betaine, proline, and trehalose (Tr). Compatible solutes can stabilize proteins and nucleic acids and support enzyme function under adverse conditions (15–19). In lactic acid bacteria, glycine betaine uptake is mediated by dedicated transport systems whose expression and transport activity can be osmotically regulated, and evidence also indicates that betaine transport activity can vary with growth temperature, consistent with an environmentally tuned acquisition route under stress (20, 21). For trehalose, increasing trehalose availability in *Lactococcus lactis* improves survivability under both acid shock and cold shock, demonstrating that trehalose can causally enhance tolerance under combined stresses rather than acting as an inert additive (22).

Although compatible solutes and cold-adapted lactic acid bacteria have been explored in food and fermentation contexts, their regulatory roles in silage environments remain insufficiently resolved, particularly under the realistic dual constraints of low temperature and low pH. Moreover, in many silage additive studies, treatment performance is still judged primarily by fermentation endpoints such as pH, organic acids, ammonia nitrogen, and viable counts, while mechanistic readouts of the cold shock axis and evidence for cross-temperature consistency are often limited (9). Here, we address these gaps by coupling a cold shock axis readout to strain physiology and silage outcomes. We cloned the cold-response gene *CspP* and quantified its expression dynamics by RT-qPCR to connect compatible solute modulation with growth and acidification under cold stress. We then evaluated the optimal compatible solute strategy in oat silage across a realistic low-temperature gradient to test cross-temperature consistency in a silage matrix. Together, this gene-to-phenotype and cross-temperature design provides a mechanistic basis for compatible solute regulation in low-temperature silage fermentation and moves beyond incremental optimization based on endpoints alone.

## MATERIALS AND METHODS

### Fermentation and sample collection

*Pediococcus pentosaceus* OL77 was cryopreserved at the Pratacultural College, Gansu Agricultural University (Lanzhou, China) (23). The strain was revived as follows: frozen cells were rapidly thawed under running water and inoculated into liquid de Man, Rogosa, and Sharpe (MRS) broth (prepared according to the manufacturer’s instructions; pH 6.2 ± 0.2) for activation (37°C, 24 h). Cultures were subcultured twice, harvested by centrifugation, and resuspended in sterile saline. The cell suspension was adjusted to 10 (5) CFU mL^−1^ by turbidimetry for subsequent use.

Analysis of *CspP* expressions at different temperatures: liquid MRS broth (pH 6.2 ± 0.2) was inoculated with 3% (vol/vol) of the activated OL77 culture and incubated at 37°C for 1 h to standardize physiological state before cold shift. The culture was then split into three aliquots and transferred to incubators set at 25°C, 15°C, or 5°C. For each temperature, independent tubes were prepared for each sampling time point (0, 1, 3, 6, 12, and 24 h) and each biological replicate (*n* = 3). The 0 h sample was collected immediately after transfer. Cells from each of the six time points at each temperature were harvested for RNA extraction and relative *CspP* quantification.

Effect of compatible solutes on low-temperature growth of LAB: activated OL77 cells were inoculated into liquid MRS broth at 3% (vol/vol). Betaine, proline, trehalose, or sorbitol was supplemented individually to a final concentration of 0.01 mol L^−1^; the no-solute medium served as the control (CK). Cultures were incubated statically at 5°C for 30 h. Culture pH and OD_600_ were recorded at 0 h (immediately after inoculation) and every 5 h thereafter to quantify acidification and growth kinetics. For OD_600_ determination, the corresponding uninoculated medium containing the same solute concentration was used as the blank.

**Fig 1:**
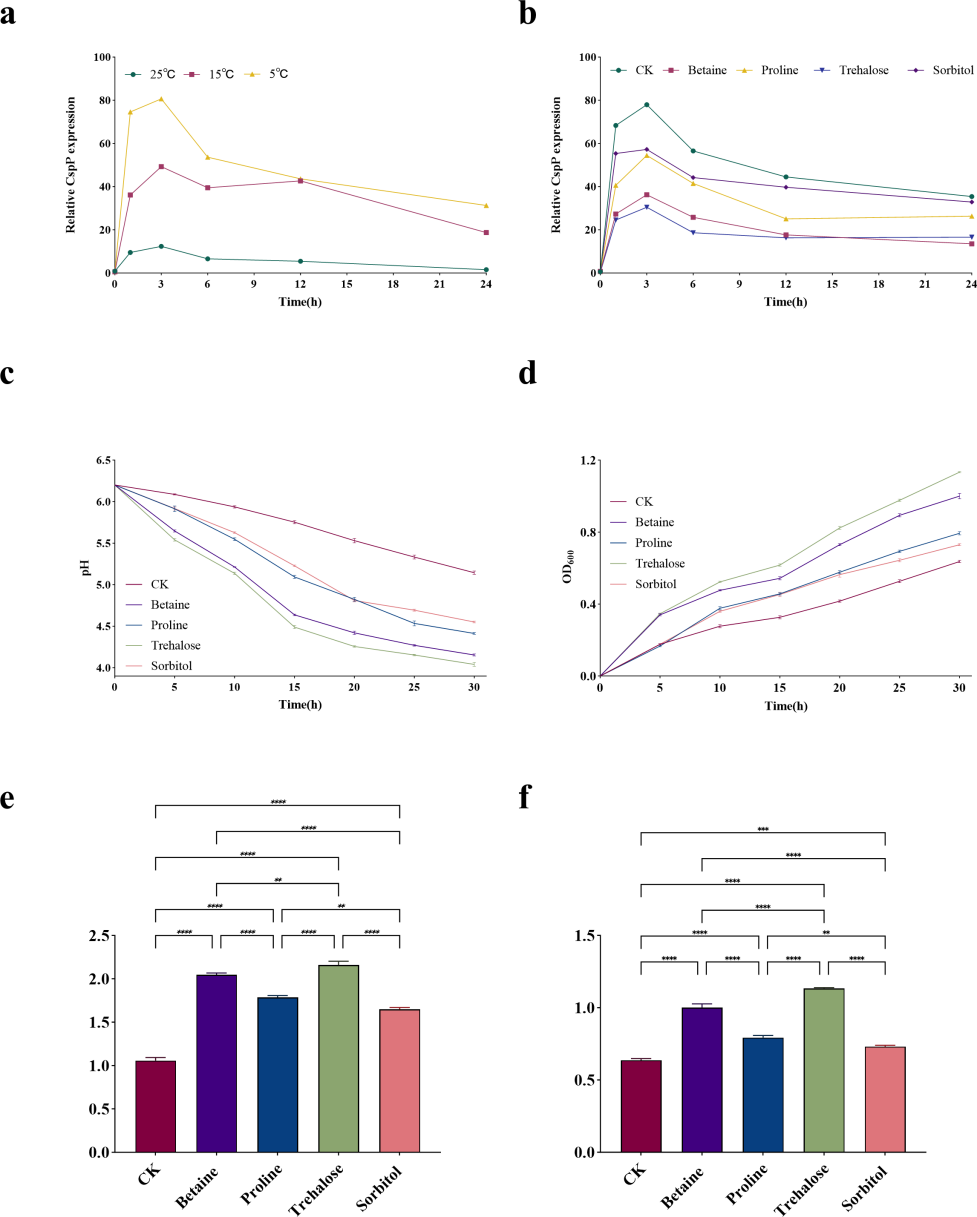
*CspP*-mediated physiological dynamic response and compatible solute regulation of *Pediococcus pentosaceus* OL77 under low-temperature stress. (**a**) Effect of low temperature on the expression of *CspP* of *Pediococcus pentosaceus* OL77. (**b**) Regulatory effects of exogenous compatible solutes on *CspP* gene expression. (**c**) Regulatory effects of compatible solutes on the acid production kinetics of strains at low temperatures. (**d**) Synergistic effects of compatible solutes on the growth rate of strains at low temperatures. Changes in acid production (**e**) and growth rate (**f**) of lactic acid bacteria throughout their growth period. *P < 0.05, **P < 0.01, ***P < 0.001, and ****P < 0.0001.

Analysis of CspP expression in response to compatible solutes: based on the temperature-screening assay (Fig. 1a), 5°C elicited the strongest *CspP* induction and was therefore selected as a stringent condition to test solute-mediated modulation. Liquid MRS broth supplemented with betaine, proline, trehalose, or sorbitol (each at 0.01 mol L^−1^) or no solute (CK) was inoculated with 3% (vol/vol) activated culture, incubated at 37°C for 1 h, and then transferred to 5°C. Samples were collected at 0, 1, 3, 6, 12, and 24 h (*n* = 3 biological replicates per time point). Cells from each of the six time points were harvested for RT-qPCR quantification of *CspP* transcripts.

### CspP cloning and expression

Total RNA was extracted using the RNAsimple Total RNA Kit (Tiangen, Beijing, China). Purity was assessed on a NanoDrop 2000 spectrophotometer (Thermo Fisher Scientific, USA); only samples with an A260/A280 ratio of 1.8–2.2 and an A260/A230 ratio of >2.0 were retained. One microgram of RNA was reverse-transcribed into 20 µL of cDNA with the PrimeScript RT Reagent Kit with gDNA Eraser (Takara, Dalian, China), diluted 1:10, and stored at −20°C.

Preliminary analyses assessed the stability of eight housekeeping genes (*tufA*, *rpoD*, *recA*, *ldh*, *gyrA*, *gyrB*, *GAPDH*, and *6PGDH*) with geNorm v3.5, NormFinder v0.953, and BestKeeper v1.0. Among them, *tufA* showed an *M* value below 0.5, ranking as the most stable under every condition and consequently chosen as the single internal control gene.

RT-qPCR was performed on a LightCycler 96 system (Roche, Switzerland). Each 20 µL reaction contained 2 µL diluted cDNA, 10 µL 2× SYBR Premix DimerEraser (Takara), 0.6 µL each of forward and reverse primers (10 µM), and nuclease-free water to a final volume of 20 µL. Cycling conditions were 95°C for 5 min; 40 cycles of 95°C for 10 s and 60°C for 30 s with data acquisition; followed by a melt-curve analysis from 60°C to 95°C in 0.5°C increments. No-template controls and no-reverse-transcription controls were included, and every sample was analyzed with three technical replicates. Relative transcript levels were calculated using the 2^−ΔΔCt^ method and normalized to *tufA*.

*CspP* cloning PCR was performed with degenerate primer CspP1 to amplify a 164 bp fragment; each 50 µL reaction contained 5 µL 10× buffer, 5 µL dNTPs (2 mM each), 0.5 µL Taq DNA polymerase (5 U µL^−1^, Takara), 1 µL of each primer (10 µM), 4 µL cDNA, and ddH_2_O up to 50 µL. Thermal cycling conditions were 94°C for 5 min; 30 cycles of 94°C for 30 s, 51°C for 45 s, and 72°C for 1 min; followed by 72°C for 10 min. PCR products were verified on a 1% agarose gel, purified, and sent to Shanghai Sangong Biotechnology Co. (Shanghai, China) for sequencing. Based on the obtained sequence, specific primers (*CspP2*) were designed and used for the RT-qPCR assays described above.

### Preparation of silage and composition

The study site was Huajialing Township, Tongwei County, Dingxi City, Gansu Province, China, situated at 2,353 m above sea level. Oat plants of cultivar Longyan 5 were machine-harvested at the milk-ripening stage and mechanically shredded. Five treatments were established: a CK; a commercial silage additive, Synlac I (SLI, Yaxin Biotechnology Co.); the selected *Pediococcus pentosaceus* OL77 (OL77); Tr alone; and a mixture of *Pediococcus pentosaceus* OL77 and trehalose (OL77 + Tr). All LAB inoculants were applied at 1 × 10^8^ CFU g ^−1^ of fresh matter (FM); sterile water of equal volume was added to the control. For the combined treatment, Tr (0.01 mol L^−1^) was pre-mixed with the OL77 suspension and thoroughly blended. Treated forages were mixed uniformly with their respective additives, packed into 5 L polyethylene mini-silos (3.2 kg per silo) at a packing density of 210 kg dry matter (DM) m^−3^. Each treatment was subdivided into three groups with three replicates apiece and incubated at 15°C, 10°C, or 5°C. After 60 days, silage samples were analyzed for nutrient contents, fermentation traits, and microbiological profiles.

A 20 g subsample of oat forage was suspended in 180 mL deionized water and cold-extracted at 4°C for 24 h; the extract was passed through four layers of gauze, and its pH was recorded using a PHS-3C digital pH meter (Shanghai Youke Instrument & Meter Co., People’s Republic of China). DM content was assessed by oven drying: 200 g of fresh sample was placed in an envelope, enzyme-inactivated at 105°C for 15 min, and dried at 65°C for ≥60 h to constant weight. The dried sample was ground, passed through a 40-mesh (0.425 mm) sieve, sealed in ziplock bags, and stored for routine nutrient analyses. Crude protein (CP) was quantified using the Kjeldahl nitrogen method (24). Water-soluble carbohydrates (WSCs) were determined via the anthrone colorimetric assay (25). Neutral detergent fiber (NDF) and acid detergent fiber (ADF) were measured following Van Soest et al. (26).

The numbers of lactic acid bacteria, aerobic bacteria, molds, and yeasts were determined by gradient dilution and spread plating. LAB were enumerated on MRS agar (prepared according to the manufacturer’s instructions; pH 6.2 ± 0.2). Aerobic bacteria were enumerated on standard nutrient agar (peptone 10 g, beef extract 3 g, NaCl 5 g, agar 15 g, 1 L water, pH 7.3 ± 0.1). Molds and yeasts were enumerated on Bengal red agar (peptone 5 g, glucose 10 g, KH_2_PO_4_ 1 g, Bengal red 0.033 g, MgSO_4_ 0.5 g, chloramphenicol 0.1 g, agar 18.5 g, pH 6.3 ± 0.1). Each sample was plated in triplicate.

Ammoniacal nitrogen (NH₃-N) was determined using the phenol–sodium hypochlorite colorimetric method (27). Lactic acid (LA), acetic acid (AA), propionic acid (PA), and butyric acid (BA) were analyzed on an Agilent 1260 high-performance liquid chromatography system. Chromatographic conditions were SB-AQ C18 column (4.6 mm × 250 mm); mobile phase A (methanol) to mobile phase B [0.01 mol L^−1^ (NH_4_)_2_HPO_4_, pH 2.70] at 3:97; flow rate 1 mL min^−1^; injection volume 20 µL; detection at 210 nm; and column temperature 25°C.

### Statistical analysis

Using the general linear model module of SPSS 29.0 (IBM, Armonk, NY, USA), we carried out two-factor ANOVA for indices measured under different temperatures and additives, followed by Duncan’s post hoc test to compare treatment means at *P* < 0.05. Effect size calculations were performed in R using the metafor package to quantify treatment impacts relative to the control across temperatures.

Random forest modeling (R, randomForest) was performed following Zhang et al. (28) as a feature-reduction step to identify a compact, temperature-robust indicator set from the 45-silo data set (three temperatures × five treatments × three replicates), using all measured silage parameters as predictors and treatment (CK, SLI, OL77, Tr, and OL77 + Tr) as the response, with ntree = 500 and a fixed seed. Performance was assessed by repeated 5-fold times 10-fold cross-validation; the minimum cross-validated error determined the smallest retained variable set; and variable importance was ranked by the mean decrease in accuracy.

## RESULTS

### Temperature- and compatible solute-responsive patterns of the *CspP* gene in *Pediococcus pentosaceus* OL77

Figure 1a illustrates the expression of the *CspP* gene in *Pediococcus pentosaceus* OL77 at various temperatures. As the temperature decreased, the relative transcription level of *CspP* rose sharply during the early cultivation phase (0–3 h). Compared with cells incubated 3 h at 25°C, those at 5°C showed a 6.54-fold increase in *CspP* transcript abundance. Although *CspP* expression started to decrease after 6 h, it consistently stayed higher in cultures exposed to low temperatures. Evidently, 5°C elicited the strongest *CspP* response in *P. pentosaceus* OL77. However, it remains unclear whether its expression levels change in the presence of compatible solutes. Therefore, we investigated how compatible solutes modulate *CspP* expression under 5°C conditions (Fig. 1b). Results indicated dramatic upregulation of *CspP* across all treatments; in the CK group, the rise reached 108-fold over the 0 h baseline. The second highest induction was observed in sorbitol treatment. Conversely, betaine and Tr treatments produced the lowest expression (60.01% and 64.05% below the CK, respectively). With prolonged incubation, transcription levels declined in each treatment yet remained lowest in the betaine and Tr groups.

The pH trajectories and OD_600_ readings (Fig. 1c and d) show that compatible solutes differentially modulate OL77’s acidification ability and growth kinetics. Compared with CK, every solute-amended treatment enhanced both acid production and growth of OL77 at low temperature (*P* < 0.05), although their efficacy varied. Among them, betaine and Tr exerted the most pronounced effects, markedly accelerating cellular metabolism. Over the full incubation course, Tr reduced terminal pH by 21.91% vs the control and raised OD_600_ by over twofold, underscoring its optimal role in boosting acidification and low-temperature growth. In contrast, proline and sorbitol were relatively ineffective, showing merely a slight promotion during the late phase.

### Nutritional quality analysis of silage treated with compatible solutes and lactic acid bacterial inoculants

The chemical composition and epiphytic microbial counts of the pre-ensiled oat are presented in Table 1. The fresh oat forage contained 331.7 g kg⁻¹ FM of dry matter. As the substrate for silage fermentation, the oats used in the present study had a WSC concentration of 198.5 g kg⁻¹ DM, surpassing the minimum threshold of 35 g kg⁻¹ DM required for high-quality silage, whereas the epiphytic LAB count was 4.15 log_10_ CFU g⁻¹ FM, below the minimum desirable value of 5.0 log_10_ CFU g⁻¹ FM.

**TABLE 1:** Chemical and microbial compositions of oat prior to ensiling^*a*^

Items	Oat (*n* = 3)
DM (g kg^−1^ FM)	331.7
CP (g kg^−1^ DM)	97.9
WSC (g kg^−1^ DM)	198.5
NDF (g kg^−1^ DM)	575.2
ADF (g kg^−1^ DM)	334.1
pH	6.15
LAB (log_10_ CFU g^−1^ FM)	4.13
Aerobic bacteria (log_10_ CFU g^−1^ FM)	7.56
Mold (log_10_ CFU g^−1^ FM)	3.94
Yeast (log_10_ CFU g^−1^ FM)	4.63

^*a*^ DM, dry matter; FM, fresh matter; LAB, lactic acid bacteria.

According to Table 2, fermentation temperature interacted with additive type to affect CP and WSC but exerted no interactive effect on DM, NDF, or ADF. Across all temperatures, the OL77 + Tr and OL77 treatments maintained higher DM levels, with OL77 + Tr being the most stable and exceeding the CK. By contrast, Tr applied in isolation (Tr) showed no discernible benefit. Lower temperatures significantly raised NDF, with OL77 + Tr topping the list at every temperature, while ADF responded inversely. Additive application significantly elevated the crude protein content of the resulting silage (*P* < 0.05), yet the magnitude of this effect varied with temperature. Nevertheless, OL77 + Tr did not maintain this positive effect uniformly across temperatures, and Tr alone surpassed CK only at the ultralow temperature of 5°C (*P* < 0.05). At all temperatures, CK recorded the lowest WSC content, but the difference from Tr was not significant. At 15°C, the SLI treatment produced the highest WSC level, followed by OL77 + Tr. Under 10°C and 5°C, OL77 yielded the greatest WSC contents, exceeding CK by 25.56% and 22.80%, respectively (*P* < 0.05), with OL77 + Tr coming next and not differing significantly from OL77. Overall, the OL77 and OL77 + Tr treatments exhibited superior nutrient preservation across the temperature range.

**TABLE 2:** Effect of temperature and additives on nutrients and ammonia nitrogen of oat silage^*a*^

Temperature	Additives	DM (g kg^−1^ FM)	NDF (g kg^−1^ DM)	ADF (g kg^−1^ DM)	CP (g kg^−1^ DM)	WSC (g kg^−1^ DM)
15°C	CK	297.1	51.13	37.81	86.0^c^	97.2^f^
	SLI	307.7	49.98	35.58	96.6^a^	128.0^ab^
	OL77	309	51.48	33.49	96.8^a^	123.5^bc^
	Tr	297.2	53.53	33.98	84.7^c^	100.4^ef^
	OL77 + Tr	319.2	54.28	32.16	97.6^a^	124.8^abc^
10°C	CK	300.7	52.21	36.57	66.9^f^	102.9^de^
	SLI	303.2	53.02	36.6	92.3^ab^	122.0^c^
	OL77	317.4	54.87	32.87	97.1^a^	129.2^a^
	Tr	297.6	54.08	33.34	66.0^f^	104.3^de^
	OL77 + Tr	316.2	58.97	33.27	96.6^a^	124.5^abc^
5°C	CK	297.2	52.21	36.57	71.7^e^	102.2^de^
	SLI	298.2	53.02	36.6	84.8^c^	105.2^d^
	OL77	312.1	54.87	32.87	87.9^bc^	125.6^abc^
	Tr	301.8	54.08	33.34	78.6^d^	102.6^de^
	OL77 + Tr	319.5	58.97	33.27	95.3^a^	125.5^abc^
Standard error		0.99	0.28	0.21	0.41	0.37
Significance						
Temperature		0.863	0.002	0.294	*P* < 0.001	*P* < 0.001
Additives		*P* < 0.001	*P* < 0.001	*P* < 0.001	*P* < 0.001	*P* < 0.001
T × A		0.542	0.223	0.464	*P* < 0.001	*P* < 0.001

^*a*^ A, additives; ADF, acid detergent fiber; CK, control; CP, crude protein; DM, dry matter; FM, fresh matter; NDF, neutral detergent fiber; OL77, *Pediococcus pentosaceus *OL77; OL77 + Tr, a mixture of *Pediococcus pentosaceus *OL77 and trehalose; T, temperature; Tr, trehalose; WSC, water-soluble carbohydrate. Values with different small letters show significant differences among treatments in the same column (*P *< 0.05).

### Analysis of silage fermentation quality of compatible solutes and additives

Table 3 presents the fermentation attributes of oat silage produced at various temperatures with the respective additives. During ensiling, silage pH increased as temperature declined, with the greatest rise observed in the CK and Tr treatments and a lesser increase in the SLI and OL77. In comparison, the OL77 + Tr treatment maintained pH values under 4.2 across all temperature regimes. Concentrations of LA and AA declined as temperature fell; nevertheless, OL77 + Tr yielded significantly more LA yet markedly less AA than all other treatments at each temperature (*P* < 0.05). PA and BA were elevated only in the Tr group at 15°C, without a significant difference from CK; minor amounts persisted in Tr at 10°C, whereas none were detected in any treatment at 5°C. NH₃-N concentration showed an inverse relationship to crude protein, first increasing and then declining with progressive cooling. All treatments supplemented with LAB recorded NH₃-N contents significantly below the CK (*P* < 0.05); OL77 + Tr delivered minimal NH₃-N across all thermal conditions. Moreover, at 15°C and 10°C, the Tr group showed NH₃-N levels exceeding those of the CK. Collectively, these findings indicate that OL77 + Tr markedly improves oat‐silage fermentation across all temperatures, providing superior protein preservation and organic acid metabolism.

**TABLE 3:** Effect of temperature and additives on the fermentation quality of oat silage^*a*^

Temperature	Additives	pH	LA (g kg^−1^ DM)	AA (g kg^−1^ DM)	PA (g kg^−1^ DM)	BA (g kg^−1^ DM)	NH_3_-N (g kg^−1^ TN)
15°C	CK	4.10^e^	62.5^d^	28.4^b^	8.3^a^	8.2^a^	71.1^d^
	SLI	3.86^f^	87.3^c^	31.8^a^	2.6^b^	2.6^b^	30.9f^g^
	OL77	3.71^f^	111.4^a^	22.5^de^	ND^d^	ND^d^	22.4^g^
	Tr	4.09^e^	61.9^d^	27.8^b^	8.4^a^	8.5^a^	73.0^d^
	OL77 + Tr	3.75^f^	113.2^a^	18.4^f^	ND^d^	ND^d^	18.2^g^
10°C	CK	4.71^c^	42.6^f^	24.2^c^	ND^d^	ND^d^	140.2^a^
	SLI	4.36^d^	89.7^c^	24.5^c^	ND^d^	ND^d^	37.6^ef^
	OL77	3.86^f^	101.7^b^	13.2^g^	ND^d^	ND^d^	24.9^g^
	Tr	4.69^c^	42.2^f^	24.4^c^	1.6^c^	1.9^c^	144.3^a^
	OL77 + Tr	3.76^f^	112.8^a^	17.8^f^	ND^d^	ND^d^	19.8^g^
5°C	CK	5.29^a^	21.0^h^	21.8^e^	ND^d^	ND^d^	114.3^b^
	SLI	4.92^b^	42.2^f^	21.4^e^	ND^d^	ND^d^	75.2^d^
	OL77	4.38^d^	55.2^e^	12.6^g^	ND^d^	ND^d^	43.5^e^
	Tr	5.04^b^	34.6^g^	23.6^cd^	ND^d^	ND^d^	89.2^c^
	OL77 + Tr	3.87^f^	91.4^c^	7.3^h^	ND^d^	ND^d^	17.6^g^
Standard error		0.02	0.55	0.10	0.03	0.01	1.02
Significance							
Temperature		*P* < 0.001	*P* < 0.001	*P* < 0.001	*P* < 0.001	*P* < 0.001	*P* < 0.001
Additives		*P* < 0.001	*P* < 0.001	*P* < 0.001	*P* < 0.001	*P* < 0.001	*P* < 0.001
T × A		*P* < 0.001	*P* < 0.001	*P* < 0.001	*P* < 0.001	*P* < 0.001	*P* < 0.001

^*a*^ A, additives; AA, acetic acid; BA, butyric acid; CK, control; LA, lactic acid; ND, not detected; NH3-N, ammonia nitrogen; OL77, *Pediococcus pentosaceus *OL77; OL77 + Tr, a mixture of *Pediococcus pentosaceus OL77* and trehalose; PA, propionic acid; T, temperature; TN, total nitrogen; Tr, trehalose. Values with different small letters show significant differences among treatments in the same column (*P *< 0.05). Values with different small letters show significant differences among treatments in the same column (*P *< 0.05).

### Reaction of silage microbial populations to compatible solutes and LAB inoculants

Fermentation temperature, lactic acid bacterial additives, and their interaction had a highly significant impact on the population sizes of key microbial groups in cold-stored oat silage (*P* < 0.05). LAB counts declined markedly as temperature decreased. Under 15°C, every LAB-supplemented group showed LAB counts that exceeded those of CK and Tr (*P* < 0.05); the OL77 + Tr treatment ranked first; OL77 ranked second. Even at 5°C, where LAB numbers were generally reduced, OL77 + Tr retained significantly higher LAB populations than any other treatment (*P* < 0.05). Regarding aerobic bacteria, CK always showed the highest counts at all three temperatures, whereas OL77 and OL77 + Tr remained lower, consistently below 5.10 log_10_ CFU g⁻¹ FM. Aerobic bacterial counts declined slightly with temperature, ranging overall from 5.29 to 6.03 log_10_ CFU g⁻¹ FM. The OL77 and OL77 + Tr treatments produced the lowest mold and yeast counts. No colony growth was detected for these treatments at 15°C or 10°C. At each temperature, LAB-inoculated groups exhibited mold and yeast levels significantly below those of CK (*P* < 0.05), yet the Tr treatment always retained comparatively high contaminant counts. Notably, at the low temperature of 5°C, OL77 + Tr markedly reduced mold and yeast populations. Overall, the OL77 + Tr combination consistently inhibited spoilage microorganisms at each low-temperature level, maintaining a stable microbial community and exhibiting robust antibacterial efficacy.

### Effect size analysis of different treatment methods on silage parameters

**Fig 2:**
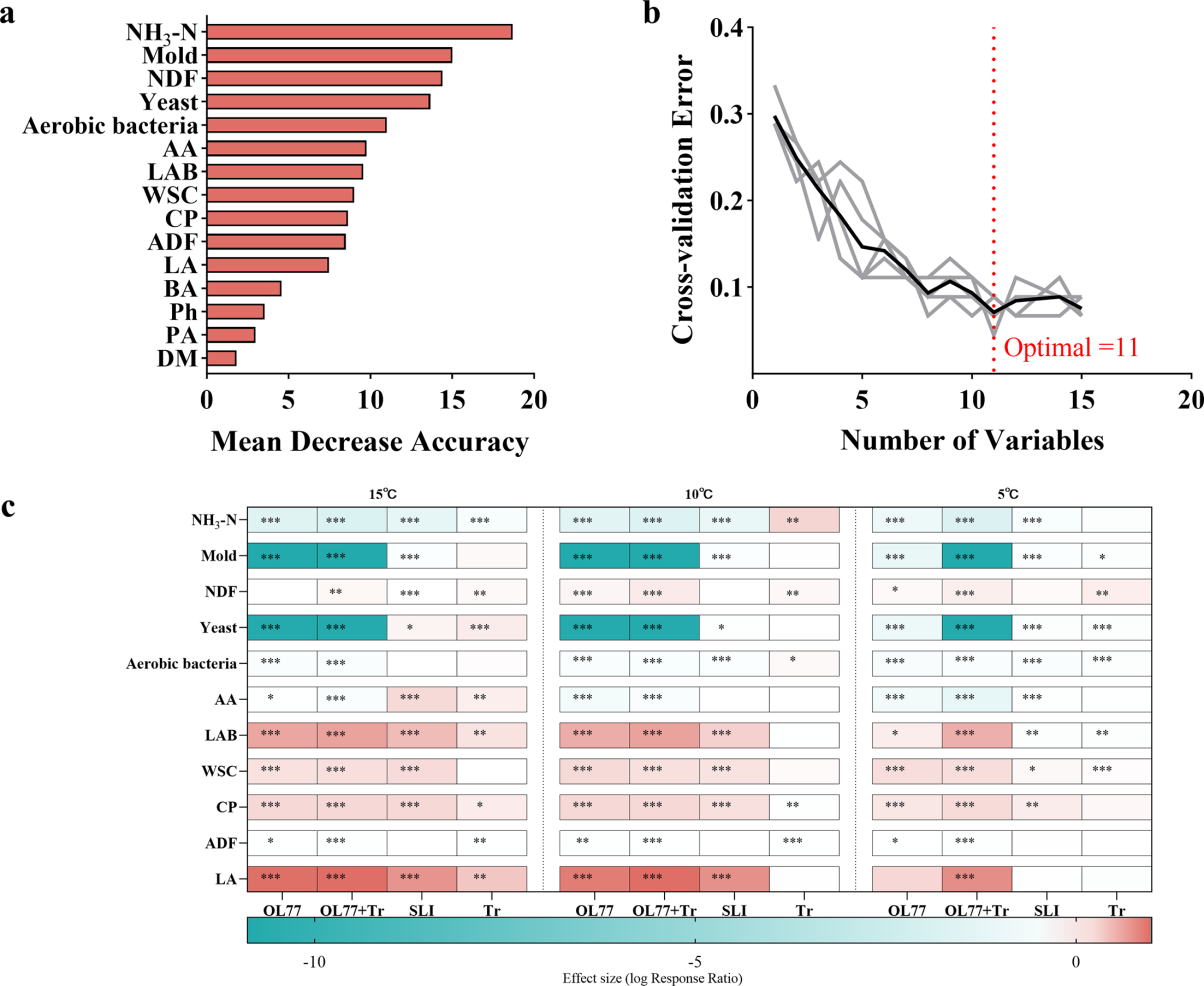
Temperature-dependent additive effects on eleven key silage quality indicators. (**a**) Ranking of variable importance for model classification accuracy based on silage parameters. (**b**) Convergence relationship between 10-fold cross-validation error and the number of input variables. (**c**) Effect size heat map of additives on 11 key silage indicators at different temperatures (vs CK). OL77, *Pediococcus pentosaceus* OL77; Tr, trehalose; OL77 + Tr, a mixture of *Pediococcus pentosaceus OL77* and trehalose. *, *P*＜0.05; **, *P*＜0.001; ***, *P*＜0.001.

Random forest analysis was used as a feature-reduction step to identify a compact set of silage-quality indicators that best discriminated against the five treatments across temperatures. In the variable importance ranking (Fig. 2a), NH₃-N and fungal loads were the strongest discriminators, with NH_3_-N, yeast count, and mold count ranked among the highest-importance variables; pH and major fermentation acids (lactic and acetic acids) also ranked highly, indicating that proteolysis control and suppression of spoilage microorganisms, together with fermentation intensity, contributed most to treatment separation. Consistent with this, cross-validation error decreased rapidly as variables were added and reached a minimum when 11 variables were included, after which additional variables provided little improvement (Fig. 2b). These 11 variables were therefore retained as a temperature-robust indicator set for summarizing treatment effects rather than inferring causality.

We then quantified the direction and magnitude of treatment effects on these indicators using standardized effect sizes relative to CK at each temperature (Fig. 2c). Across the 15°C–5°C gradient, the OL77 + Tr treatment showed the most consistent beneficial pattern, characterized by lower pH and NH₃-N, strong suppression of molds and yeasts, and higher lactic acid production, in agreement with the conventional fermentation and microbiological results in Tables 3 and 4. In contrast, Tr alone showed weaker and less consistent effects across temperatures, whereas OL77 generally improved fermentation and microbial suppression but was less stable than the combined treatment under the coldest condition. Together, Fig. 2 provides a data-driven synthesis indicating that limiting proteolysis and inhibiting fungal proliferation, alongside maintaining strong lactic fermentation, are the primary axes by which the OL77 + Tr formulation improves low-temperature oat silage quality.

**TABLE 4:** Effect of temperature and additives on microorganism quantification of oat silage (log_10_ CFU g^−1^ FM)^*a*^

Temperature	Additives	LAB	Aerobic bacteria	Mold	Yeast
15°C	CK	6.28^e^	6.91^a^	5.42^a^	5.68^a^
	SLI	8.13^c^	6.55^b^	4.31^b^	5.34^b^
	OL77	9.44^a^	5.09^d^	0^d^	0^i^
	Tr	6.22^e^	6.85^a^	5.52^a^	5.62^a^
	OL77 + Tr	9.72^a^	4.77^ef^	0^d^	0^i^
10°C	CK	4.96^g^	6.93^a^	5.29^a^	4.65^d^
	SLI	7.05^d^	5.36^c^	4.24^b^	4.47^e^
	OL77	9.06^b^	4.78^ef^	0^d^	0^i^
	Tr	4.86^g^	7.00^a^	5.35^a^	4.76^c^
	OL77 + Tr	9.68^a^	4.65^ef^	0^d^	0^i^
5°C	CK	4.24^h^	6.35^b^	5.29^a^	4.51^e^
	SLI	4.42^h^	5.09^d^	4.23^b^	4.09^g^
	OL77	5.81^f^	4.55^f^	1.85^c^	1.96^h^
	Tr	4.33^h^	5.59^c^	4.50^b^	4.22^f^
	OL77 + Tr	8.87^b^	4.89^de^	0^d^	0^i^
Standard error		0.03	0.02	0.03	0.01
Significance					
Temperature		*P* < 0.001	*P* < 0.001	*P* < 0.001	*P* < 0.001
Additives		*P* < 0.001	*P* < 0.001	*P* < 0.001	*P* < 0.001
T × A		*P* < 0.001	*P* < 0.001	*P* < 0.001	*P* < 0.001

^*a*^ A, additives; CK, control; LAB, lactic acid bacteria; OL77, *Pediococcus pentosaceus *OL77; OL77 + Tr, a mixture of *Pediococcus pentosaceus OL77 *and trehalose; T, temperature; Tr, trehalose. Values with different small letters show significant differences among treatments in the same column (*P *< 0.05).

## DISCUSSION

### Compatible solute-mediated cold adaptation of *P. pentosaceus* OL77

Low temperature is a major barrier for rapid acidification during ensiling because it suppresses lactic acid bacterial metabolism and delays the decline in pH. In the broader cold adaptation framework, cold shock proteins are rapidly induced after temperature downshifts and function as RNA chaperones that sustain translation and coordinated stress programs at low temperature (10, 11). Importantly, genetic evidence in *Lactococcus lactis* demonstrates that csp genes contribute to cold-associated phenotypes and to the production of cold-induced proteins, supporting the use of csp transcriptional dynamics as a mechanistic readout rather than a descriptive marker (14).

Consistent with this mechanistic expectation, we observed an immediate multifold induction of *CspP* transcripts in *P. pentosaceus* OL77 at 5°C, confirming rapid activation of the cold shock axis under the combined constraints of cold and progressive acidification. Betaine or Tr supplementation markedly reduced the later *CspP* peak while simultaneously increasing biomass accumulation and accelerating acidification. These coupled shifts demonstrate that compatible solutes did not merely correlate with improved fermentation but also actively reduced the cold-induced regulatory burden, enabling OL77 to sustain growth and lactic acid production at low temperature. A plausible basis is that compatible solutes stabilize ribosomes and enzymes and support membrane-associated functions under stress, which can lessen reliance on strong *Csp* induction (10, 29). For betaine, this interpretation is supported by evidence that betaine transport activity in lactic acid bacteria responds not only to osmotic upshift but also to growth temperature, implying an environmentally tuned uptake route under stress (20). For Tr, independent work in *Lactococcus lactis* has shown that increasing trehalose availability enhances survival under both acid shock and cold shock, consistent with a causal protective role under combined stress conditions (22). In contrast, proline and sorbitol provided delayed protection in our culture assays, consistent with slower uptake or lower immediate intracellular availability under acute cold shock. These differences indicate that compatible solute efficacy depends on both chemical properties and metabolic accessibility, and they identify Tr (and betaine) as the most effective regulators of OL77 cold performance at the strain level. Building on this mechanistic and physiological evidence, we next tested whether the optimal formulation translates into improved fermentation control in oat silage across a realistic low-temperature gradient (5°C–15°C). Future work should map transport and utilization routes in OL77 and quantify intracellular solute dynamics to further resolve how compatible solutes couple to the cold shock network.

### Tr-assisted OL77 improves fermentation under suboptimal temperatures

WSC and the abundance of LAB are the two fundamental determinants of successful silage fermentation (30). Despite the high WSC level in the raw oat material (198.5 g kg⁻¹ DM), the naturally adherent LAB population (4.15 log_10_ CFU g⁻¹ FM) was below the critical limit for reliable fermentation (31), making external LAB inoculation essential. As the dominant strain selected in this study, OL77 exhibited strong acid-producing capacity and broad temperature adaptability. Across the 15°C–5°C gradient, the OL77 + Tr treatment markedly increased lactic acid concentration, kept pH below 4.2 even at 5°C, and significantly curtailed NH₃-N accumulation. Importantly, this formulation was evaluated across a realistic low-temperature range rather than at a single temperature, and OL77 + Tr showed consistently superior fermentation performance throughout 5°C–15°C, supporting cross-temperature robustness for cold-season and high-altitude ensiling. The result suggests that OL77 plus Tr possesses strong fermentative propulsion in low-temperature settings.

The augmented performance likely results from two functions of Tr: (i) acting as a non-reducing compatible solute, it stabilizes bacterial membranes and glycolytic enzymes through the water-replacement mechanism, thus improving physiological resistance to low-temperature stress (32); (ii) Tr may also provide readily available carbohydrates that support early-stage fermentation in strains capable of utilizing it, thereby shortening the acidification lag and promoting rapid lactic acid accumulation. Notably, our strain-level assays showed that Tr supplementation enhanced growth and acidification under cold stress, supporting a physiological basis for improved fermentation start-up. However, Tr alone displayed relatively inconsistent performance. While Tr reduced NH₃-N relative to the control at 5°C, it elevated NH₃-N at 10°C–15°C and showed uneven protection of DM and CP. These results suggest that Tr requires synergy with an adapted inoculant to translate stress protection into stable fermentation control, rather than acting as a universally effective additive on its own.

Temperature is the dominant factor governing the rate of silage fermentation and the succession of microbial communities. When the temperature drops, pH tends to increase and levels of organic acids—including LA and AA decrease—a pattern largely attributable to low-temperature suppression of microbial activity and acid biosynthesis (33). Cold-induced suppression of microbial metabolism hampers acid production, slows pH reduction, and fails to curb proteolytic clostridia, which continue degrading protein (34). This explains why decreasing temperature in the present study was accompanied by higher NH₃-N. However, the increase in NH₃-N level ceased at 10°C. This is related to the fact that most clostridia (protein-degrading clostridia) remain active only at high temperatures (35). Low temperature (5°C) suppresses the activity of proteolytic *Clostridia*, limiting further protein degradation and thus slowing the accumulation of NH₃-N. Even though the addition of Tr showed marginally higher NH₃-N than both of SLI and OL77 at 5°C, it nevertheless retained appreciable microbial activity, implying that Tr may confer a broad, non-specific protection to microbial communities. Notably, the OL77 + Tr combination consistently maintained stable, high-quality fermentation throughout the entire 5°C–15°C range, demonstrating a synergistic capacity to break through the low-temperature “start-up threshold” and drive lactic fermentation forward.

Overall, Tr should ideally be used in concert with cold-adapted LAB, thereby combining its osmoprotective benefits with strain-mediated metabolism to jump-start fermentation. This “compatible solute + psychrotrophic inoculant” strategy offers an effective solution for breaking the low-temperature silage bottleneck of slow initiation and inadequate acidification.

### Synergistic effect of the OL77 + Tr combination on suppressing proteolysis and fungal proliferation during low-temperature silage

Among the 11 core indicators selected by random forest screening, protein degradation (NH₃-N) and fungal amplification were highlighted as having a decisive effect on silage quality. This finding is consistent with previous statistical conclusions on key factors in silage deterioration (36, 37). Notably, the weights assigned to NH₃-N, mold, and yeast are significantly higher than those of traditional nutritional indicators, showing that “inhibiting protein hydrolysis and controlling fungal proliferation” should be the primary objective when evaluating low-temperature silage. Correspondingly, OL77 alone or in combination with Tr significantly reduced mold and yeast content and simultaneously reduced NH₃-N in a 10°C–15°C environment, indicating that this strain effectively weakened the reproduction of harmful fungi through rapid acid production and competitive exclusion, while Tr, as an osmotic protectant and fermentable carbon source, further enhanced the metabolic activity and low-temperature adaptability of OL77. When the temperature dropped to 5°C, overall fermentation kinetics slowed markedly, and the inhibitory effect of OL77 alone weakened accordingly. At this point, Tr synergy became prominent: by stabilizing membranes and modulating cold shock proteins, it alleviates cold inhibition of LAB physiology, thereby sustaining rapid lactic acid buildup and continuously suppressing aerobes and fungi (22). The SLI treatment performed mediocrely under the same conditions, probably because the dominant microbes in this commercial inoculant are not strictly psychrophilic and cannot swiftly capture the niche at very low temperatures. Overall, the OL77 + Tr duo constitutes an efficient strategy for silage in cold regions or winter, sustaining stable quality enhancement across a wider temperature span.

The indicator set derived from random forest should be interpreted as a data-driven summary of multivariate quality changes. Given the factorial but moderate sample size, we used repeated cross-validation and limited the goal of the model to feature reduction, and we interpreted the selected indicators alongside conventional statistics and standardized effect sizes. External validation in larger field data sets will be necessary before extending the model to predictive use across new sites, years, or forage types.

### Conclusion

Supplementation of compatible solutes, particularly trehalose, synergistically enhances the cold tolerance and fermentation performance of *Pediococcus pentosaceus* OL77 under suboptimal temperatures. Trehalose mitigated cold-induced stress by promoting cell growth, accelerating acid production, and stabilizing the expression of the cold shock protein gene *CspP*. When applied to oat silage, the OL77 + trehalose combination maintained superior fermentation quality across 5°C–15°C, effectively suppressing undesirable microbes and minimizing protein degradation. In addition, the temperature-robust indicator set identified by feature-reduction analysis provides a practical basis for streamlined quality monitoring under low-temperature ensiling conditions.

## Data Availability

The 16S rRNA gene sequence of *Pediococcus pentosaceus* OL77 has been deposited in GenBank under accession number MT359243.1.
